# Supplementary material for: The Effects of a Macronutrient-Based Diet and Time-Restricted Feeding (16:8) on Body Composition in Physically Active Individuals—A 14-Week Randomised Controlled Trial
Source: Nutrients. 2021 Sep 6;13(9):3122. doi: 10.3390/nu13093122 (PMC8465090; doi:10.3390/nu13093122)
Supplement: Supplementary file 1 [file nutrients-13-03122-s001.zip › nutrients-1358966-supplementary.pdf]

**Supplemental Material**

**Figure S1 A-L**

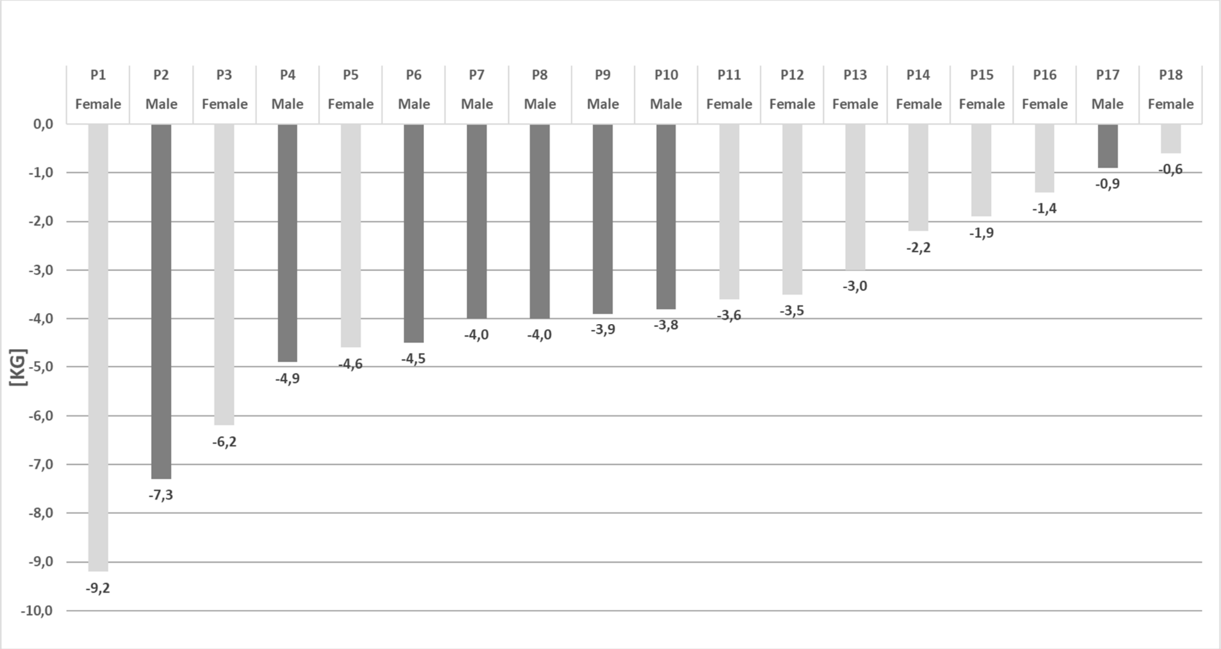


**Figure S1A: Individual changes in body weight, T0 to T2 – TRF**

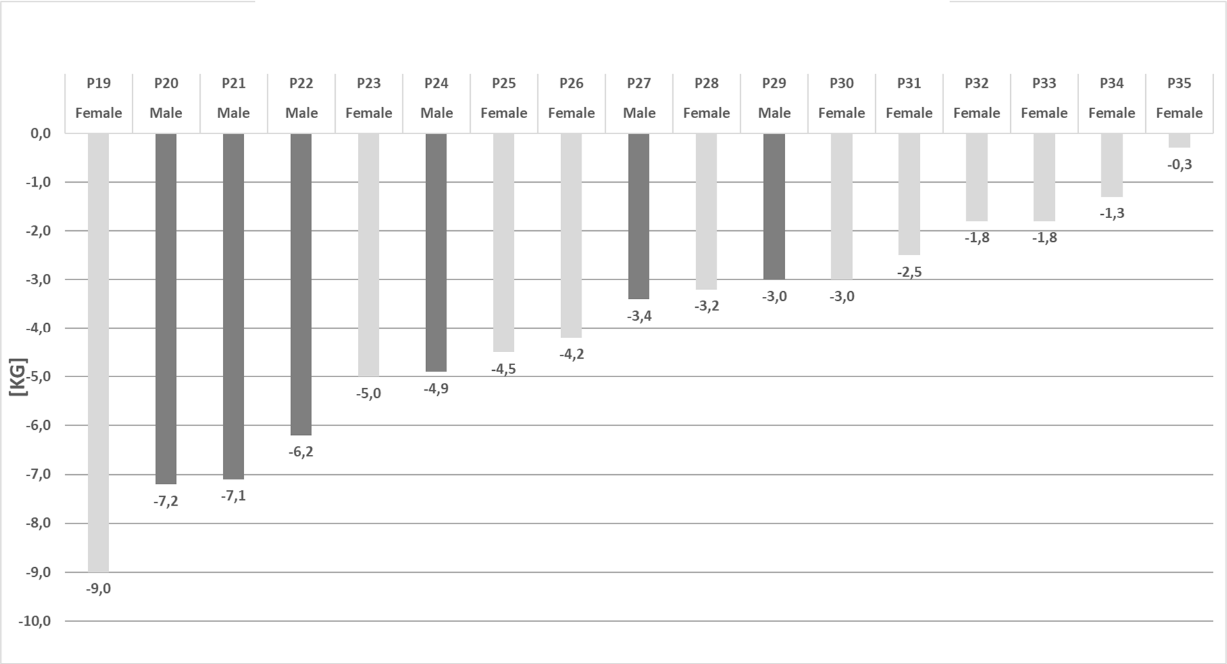


**Figure S1B: Individual changes in body weight, T0 to T2 – MBD**

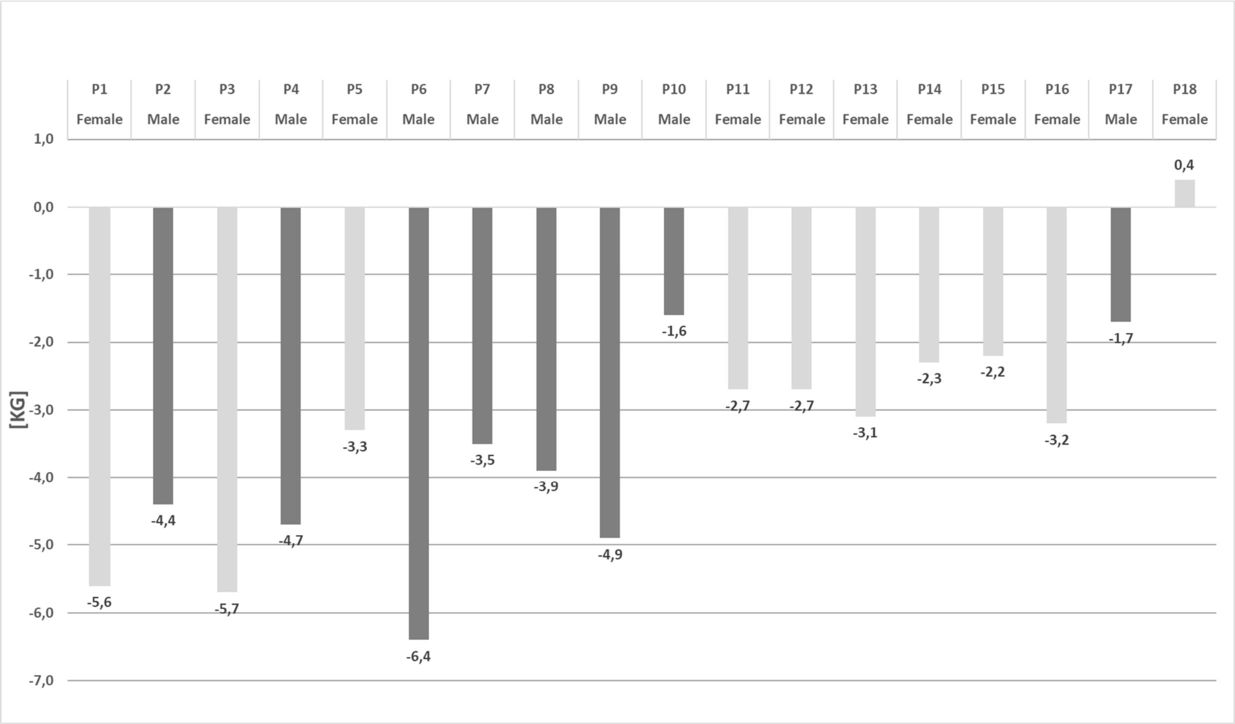


**Figure S1C: Individual changes in fat mass, T0 to T2 – TRF**

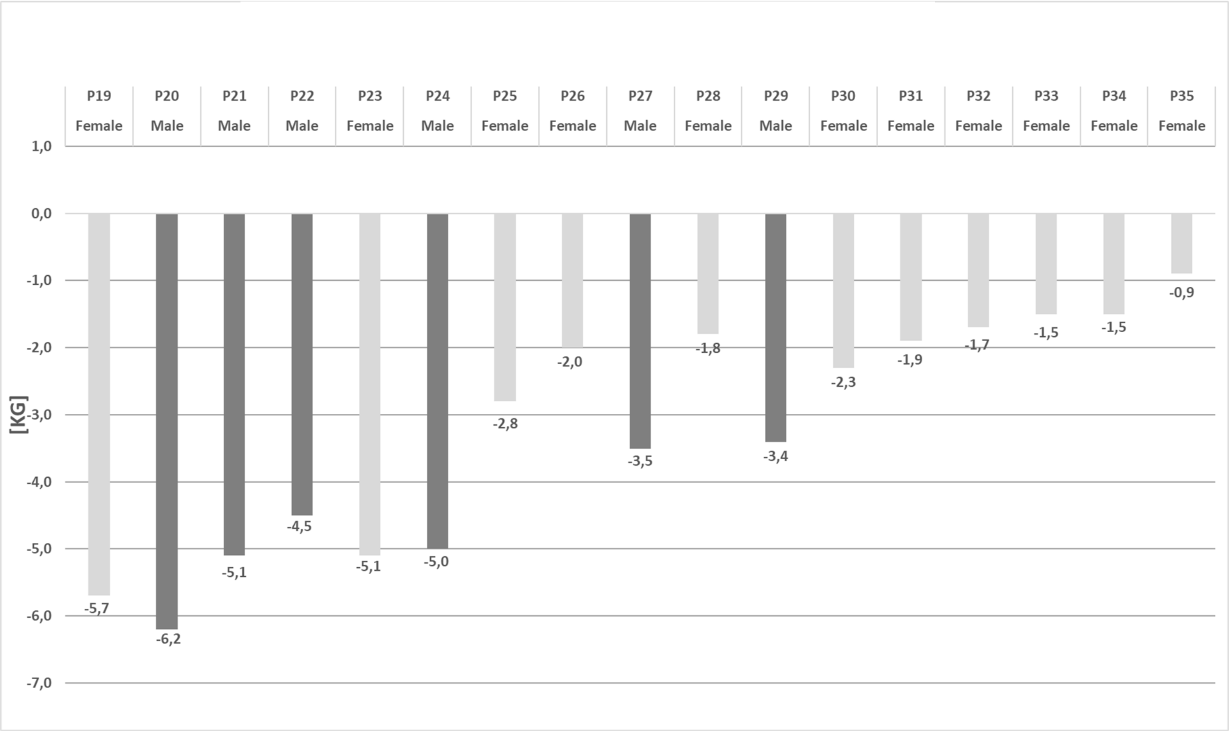


**Figure S1D: Individual changes in fat mass, T0 to T2 – MBD**

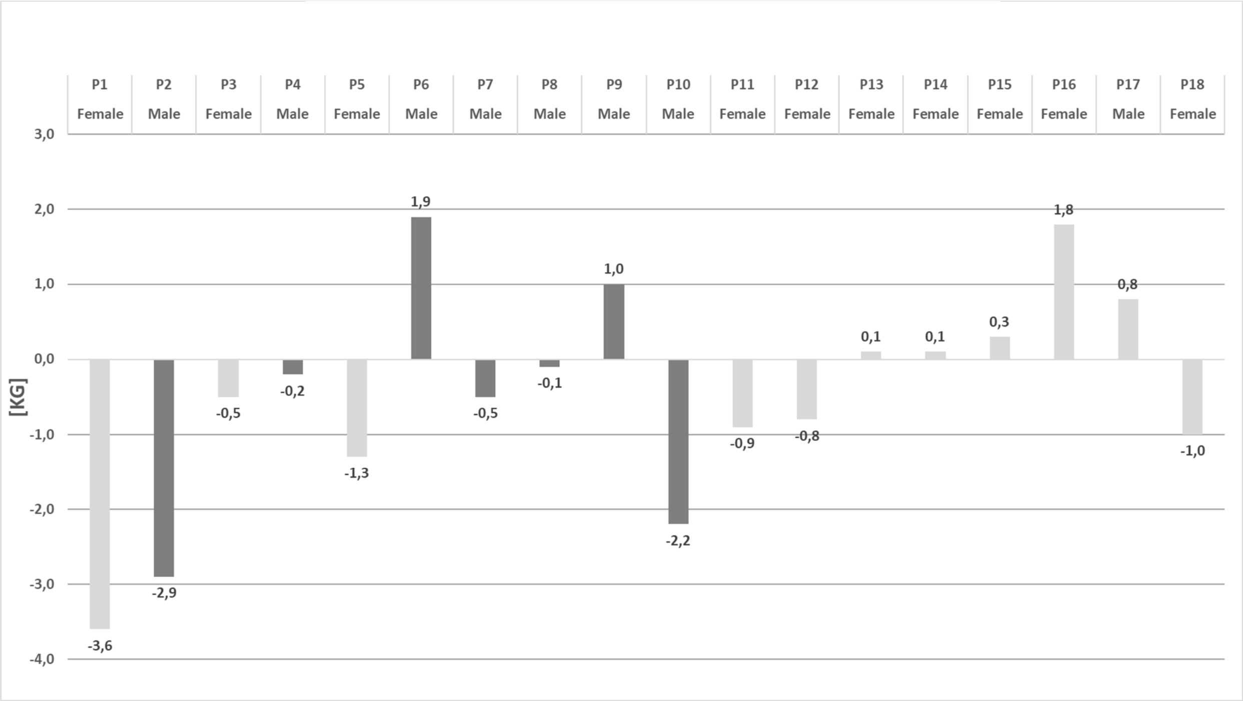


**Figure S1E: Individual changes in lean body mass, T0 to T2 – TRF**

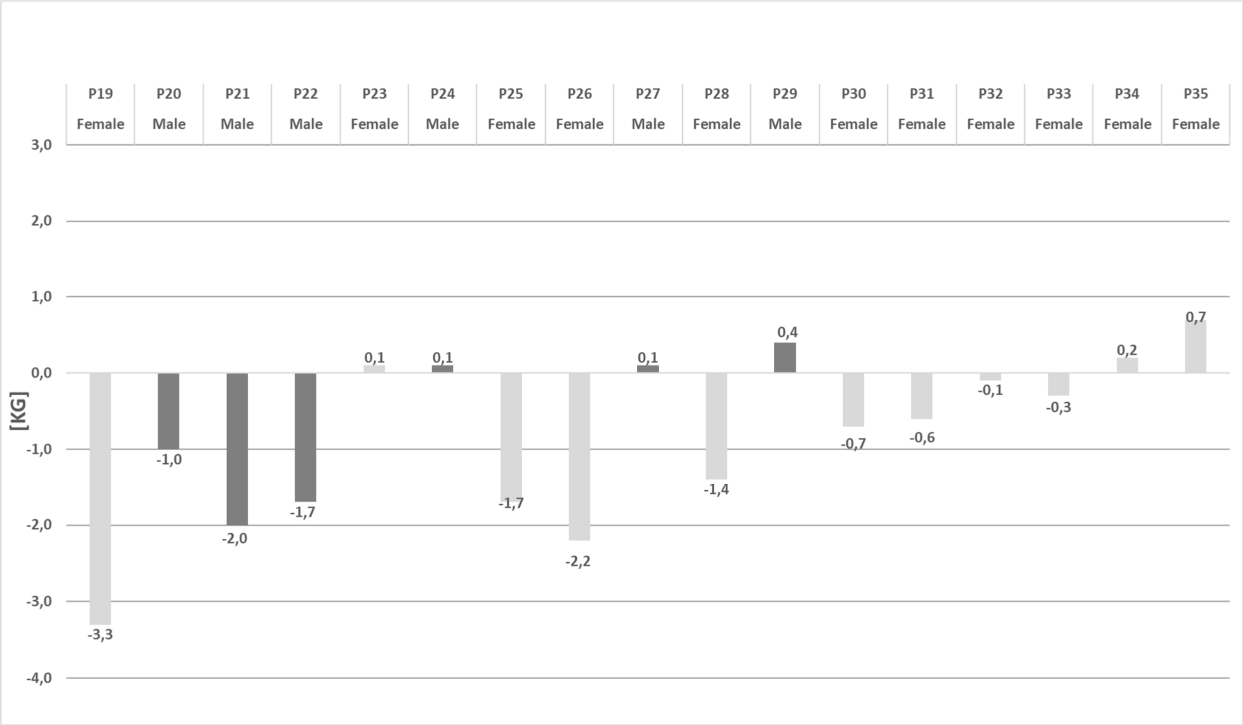


**Figure S1F: Individual changes in lean body mass, T0 to T2 – MBD**

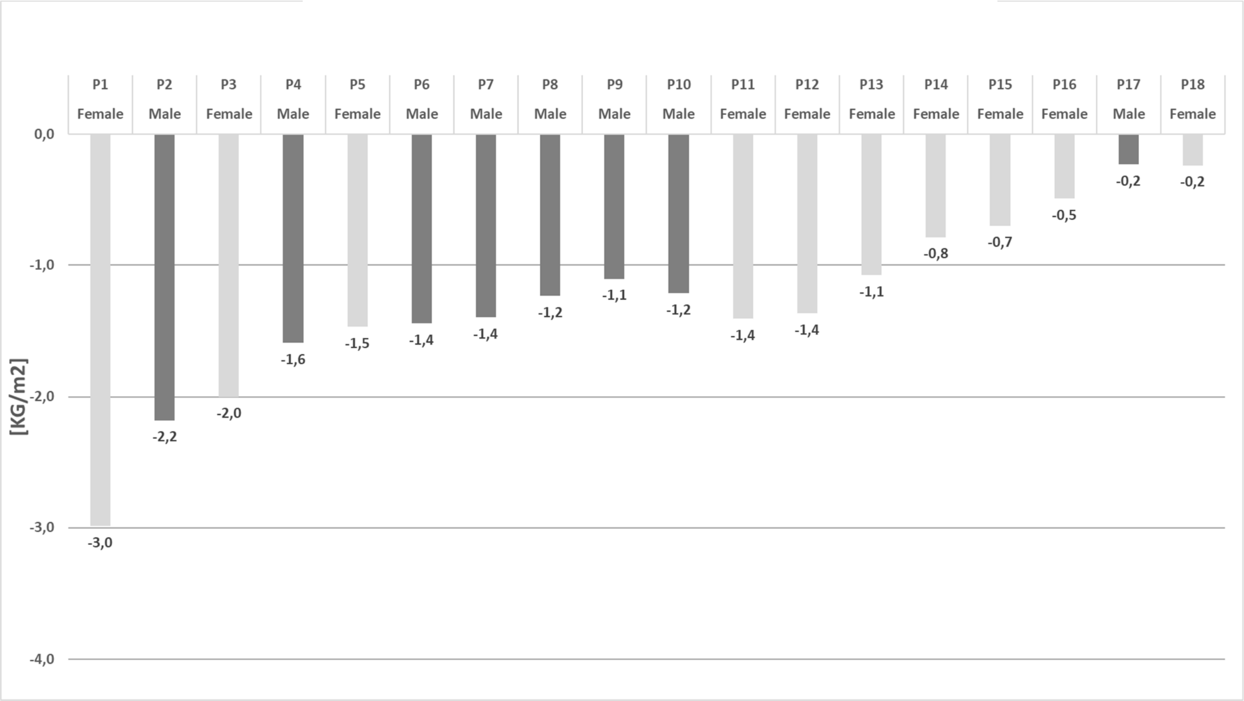


**Figure S1G: Individual changes in body mass index, T0 to T2 – TRF**

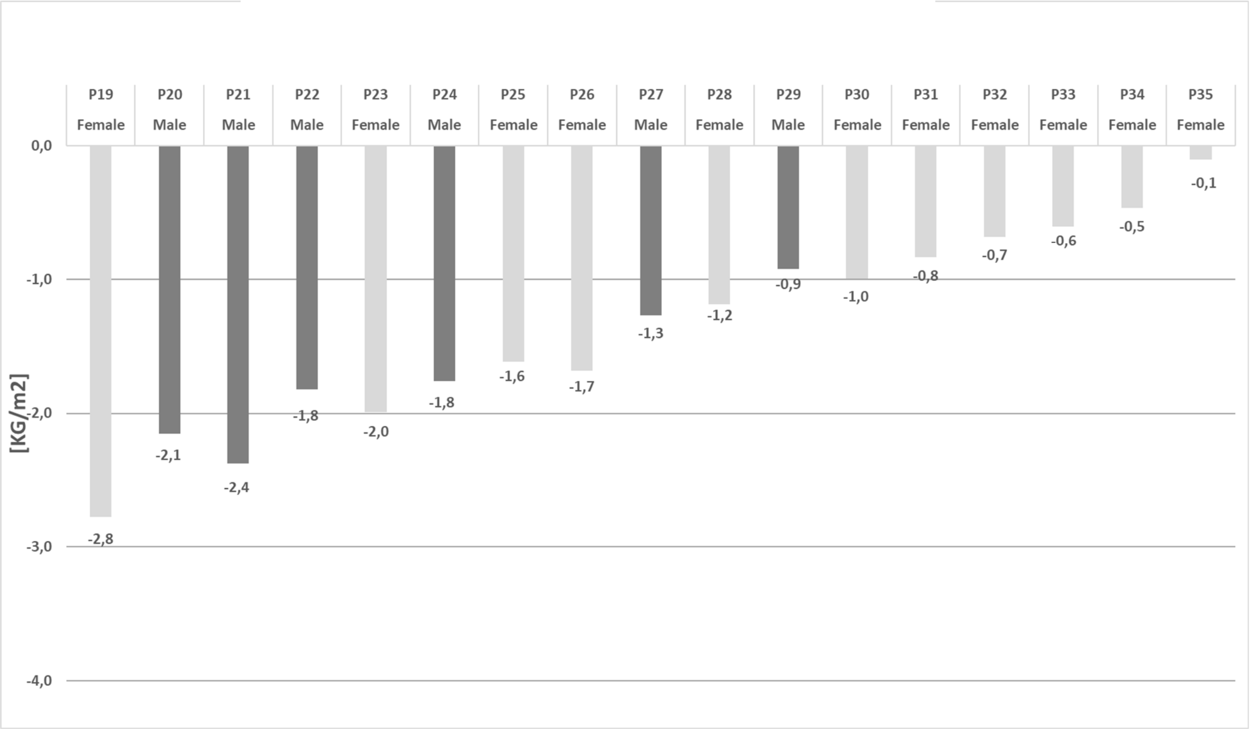


**Figure S1H: Individual changes in body mass index, T0 to T2 – MBD**

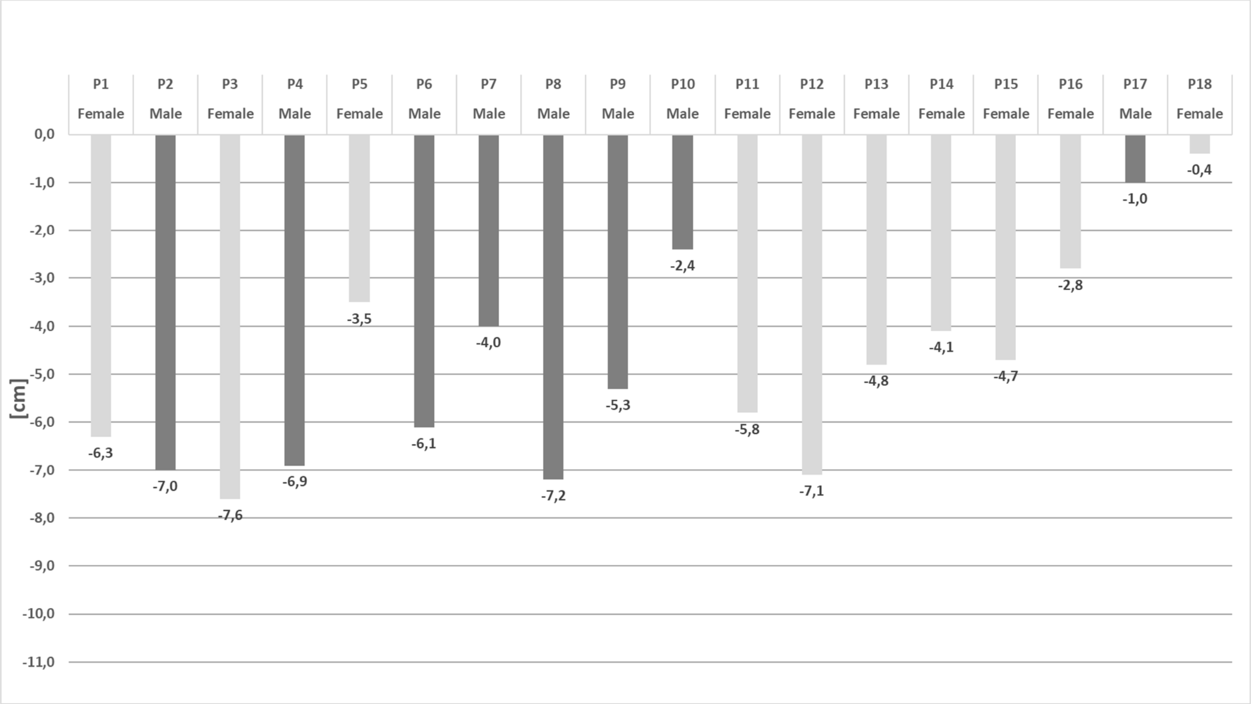


**Figure S1I: Individual changes in waist circumference, T0 to T2 – TRF**

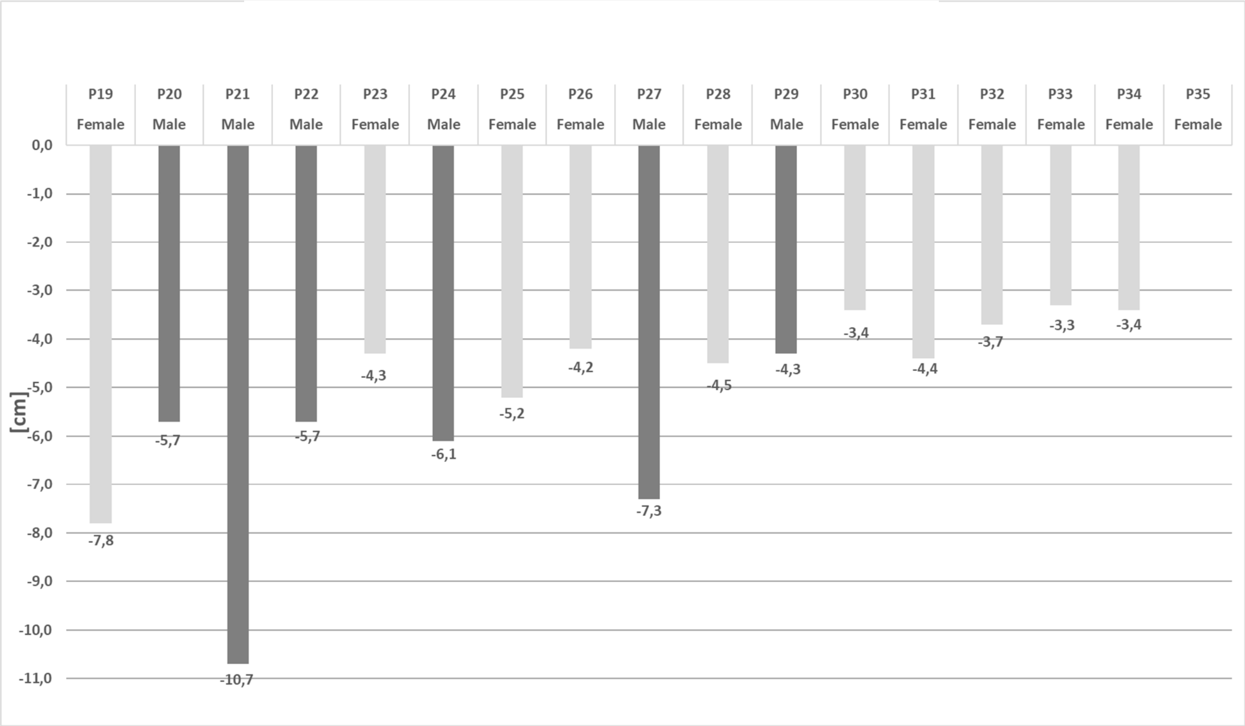


**Figure S1J: Individual changes in waist circumference, T0 to T2 – MBD**

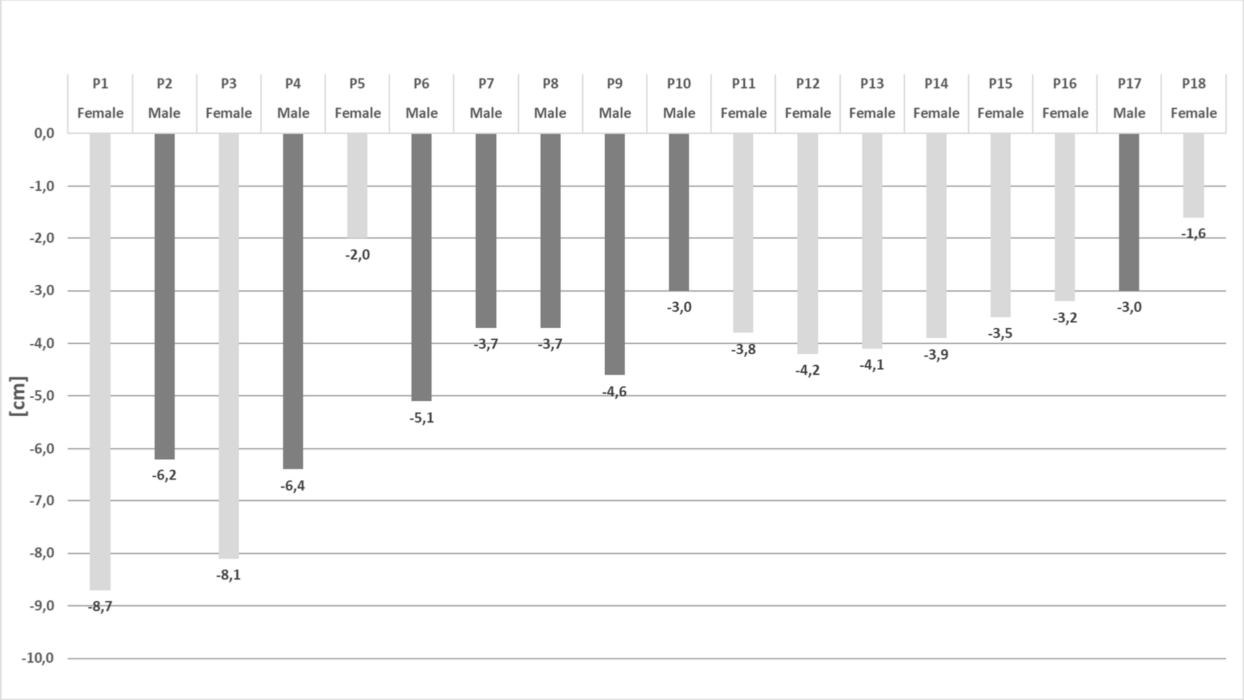


**Figure S1K: Individual changes in hip circumference, T0 to T2 – TRF**

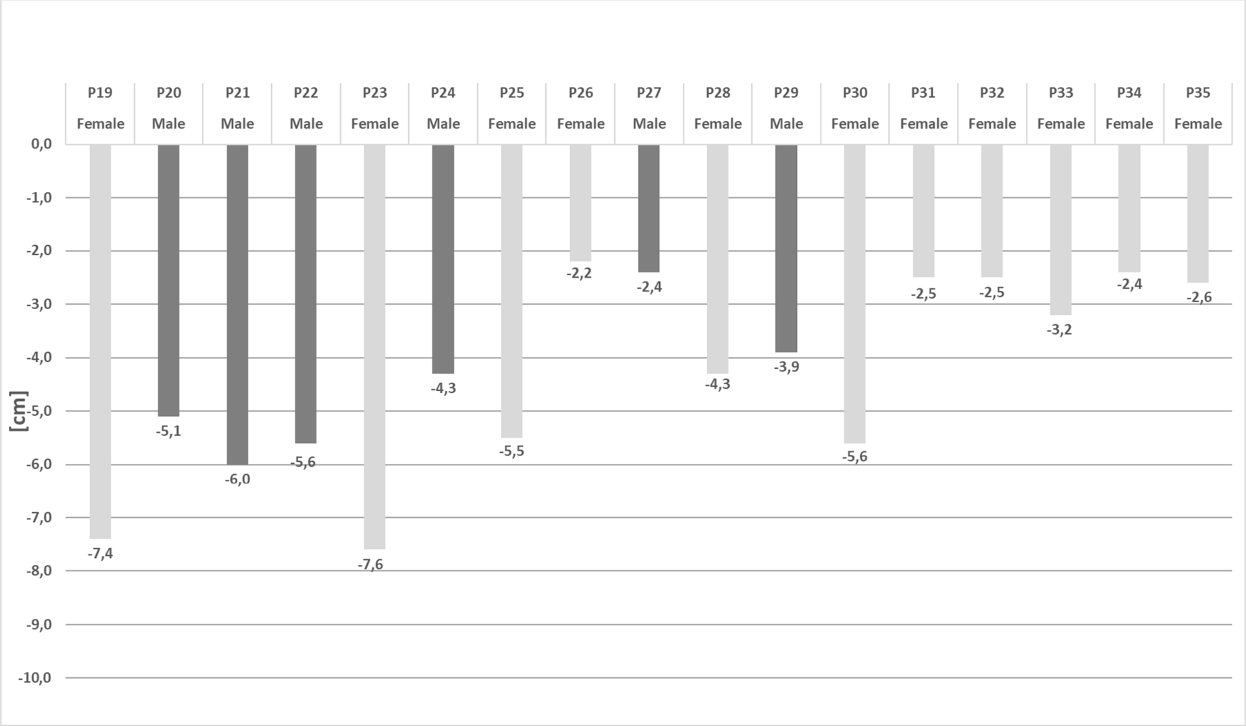


**Figure S1L: Individual changes in hip circumference, T0 to T2 – MBD**
